# Different fatty acid metabolism effects of (−)-Epigallocatechin-3-Gallate and C75 in Adenocarcinoma lung cancer

**DOI:** 10.1186/1471-2407-12-280

**Published:** 2012-07-06

**Authors:** Joana Relat, Adriana Blancafort, Glòria Oliveras, Sílvia Cufí, Diego Haro, Pedro F Marrero, Teresa Puig

**Affiliations:** 1 Biochemistry and Molecular Biology, School of Pharmacy and Institute of Biomedicine of the University of Barcelona (IBUB), 08028, Barcelona, Spain; 2 Molecular Oncology (NEOMA), School of Medicine, University of Girona and Girona Institute for Biomedical Research (IDIBGi), 17071, Girona, Spain; 3 Molecular Oncology, Catalan Institute of Oncology (ICO), Girona Institute for Biomedical Research (IDIBGi), 17007, Girona, Spain

**Keywords:** Lung cancer, Xenograft, Fatty acid synthase, EGCG, C75, Inhibitors, Weight loss, Fatty acid metabolism, EGFR

## Abstract

**Background:**

Fatty acid synthase (FASN) is overexpressed and hyperactivated in several human carcinomas, including lung cancer. We characterize and compare the anti-cancer effects of the FASN inhibitors C75 and (−)-epigallocatechin-3-gallate (EGCG) in a lung cancer model.

**Methods:**

We evaluated *in vitro* the effects of C75 and EGCG on fatty acid metabolism (FASN and CPT enzymes), cellular proliferation, apoptosis and cell signaling (EGFR, ERK1/2, AKT and mTOR) in human A549 lung carcinoma cells. *In vivo*, we evaluated their anti-tumour activity and their effect on body weight in a mice model of human adenocarcinoma xenograft.

**Results:**

C75 and EGCG had comparable effects in blocking FASN activity (96,9% and 89,3% of inhibition, respectively). In contrast, EGCG had either no significant effect in CPT activity, the rate-limiting enzyme of fatty acid β-oxidation, while C75 stimulated CPT up to 130%. Treating lung cancer cells with EGCG or C75 induced apoptosis and affected EGFR-signaling. While EGCG abolished p-EGFR, p-AKT, p-ERK1/2 and p-mTOR, C75 was less active in decreasing the levels of EGFR and p-AKT. *In vivo*, EGCG and C75 blocked the growth of lung cancer xenografts but C75 treatment, not EGCG, caused a marked animal weight loss.

**Conclusions:**

In lung cancer, inhibition of FASN using EGCG can be achieved without parallel stimulation of fatty acid oxidation and this effect is related mainly to EGFR signaling pathway. EGCG reduce the growth of adenocarcinoma human lung cancer xenografts without inducing body weight loss. Taken together, EGCG may be a candidate for future pre-clinical development.

## Background

Fatty acid synthase (E.C.2.3.1.85; FASN) is a homodimeric multienzymatic protein that catalyzes de novo synthesis of long-chain fatty acids from acetyl-CoA, malonyl-CoA, and NADPH precursors [1]. In most human tissues the diet supplies the fatty acids needs and FASN expression is low or undetectable. In contrast, in many human solid carcinomas, lipogenic enzymes (mainly FASN) are highly expressed [2-7] and *de novo* fatty acids biosynthesis supplies the needs of long chain fatty acids (LCFA) for energy production, protein acylation, synthesis of biological membranes, DNA synthesis and cell cycle progression among other biological processes, providing an advantage for tumour growth and progression [3-5].

FASN inhibition that blocks lipogenic pathway and impedes fatty acid synthesis, entails apoptosis in tumour cells that overexpress FASN, without affecting non-malignant cells (reviewed in ref. [8]). In this context, FASN enzyme has became a promising target for anti-cancer therapy, a putative biomarker of malignancy and an indicative of prognosis for many cancers, including lung carcinomas [5-7,9].

The oncogenic properties of FASN seem to be the result of an increased activation of HER2 and its downstream signaling cascades: phosphoinositide-3 kinase/protein kinase B/mammalian target of rapamycin (PI3K/AKT/mTOR), mitogen-activated protein kinase/extracellular signal-regulated kinase (MAPK/ERK1/2) pathways [10-18].

The use of FASN inhibition as anticancer therapy was first described with Cerulenin (a natural antibiotic from *Cephalosporium ceruleans)* that causes apoptotic cancer cell death *in vitro*[19]. More recently, C75, a synthetic analogue of cerulenin or (−)-epigallocatechin-3-gallate (EGCG), the main polyphenolic catechin of the green tea, have been identified as FASN inhibitors, able to induce apoptosis in several tumour cell lines and also to reduce the size of mammary tumours in animal models [8,20-24]. Although its selective cytotoxicity, C75 has been discarded in many cancer models due to its side effects: anorexia and body weight loss. In contrast, we have demonstrated that in SKBr3 breast cancer cells EGCG has similar effects as C75 in inhibiting FASN and it does not induce CPT activity *in vitro,* neither weight loss *in vivo*[11,25,26], opening new perspectives in the use of green tea polyphenols or its derivatives as anti-cancer drugs alone or in combination with other therapies.

Here we compare the effects of C75 and EGCG on lipogenesis (FASN activity), fatty acid oxidation (CPT activity), cellular proliferation, induction of apoptosis and cell signaling (EGFR, ERK1/2, AKT and mTOR) in A549 lung carcinoma cells. We also evaluated their anti-cancer activity and their effect on body weight with a mice model of A549 lung cancer xenograft. We examined EGCG as a potential drug for clinical development in adenocarcinoma of lung cancer that accounts for 40% of non-small-cell lung cancers (NSCLC), the most common type of lung cancer [27].

## Methods

### Cell Lines and Cell Culture

A549 lung cancer cells were obtained from the American Type Culture Collection (ATCC, Rockville, MD, USA), and were cultured in Dulbecco’s Modified Eagle’s Medium (DMEM, Gibco, Berlin, Germany) containing 10% heat-inactivated fetal bovine serum (FBS, HyClone Laboratories, Utah, USA), 1% L-glutamine, 1% sodium pyruvate, 50 U/mL penicillin, and 50 μg/mL streptomycin (Gibco). Cells were routinely incubated at 37 °C in a humidified atmosphere of 95% air and 5% CO_2_.

### Growth Inhibition Assay

EGCG, C75 and 3–4,5-dimethylthiazol-2-yl-2,5-diphenyltetrazolium bromide (MTT) were purchased from Sigma-Aldrich (St. Louis, MO, USA). Dose–response studies were done using a standard colorimetric MTT reduction assay. Briefly, cells were plated out at a density of 3 × 10^3^ cells/100 μL/well in 96-well microtiter plates. Following overnight cell adherence fresh medium along with the corresponding concentrations of EGCG and C75 were added to the culture. Following treatment, media was replaced by drug-free medium (100 μL/well) and MTT solution (10 μL of a 5 mg/mL), and incubation was prolonged for 2,5 h at 37°C. After carefully removing the supernatants, the MTT-formazan crystals formed by metabolically viable cells were dissolved in DMSO (100 μL/well) and absorbance was determined at 570 nm in a multi-well plate reader (Spectra max 340PC (380), BioNova Cientifica s.l., Madrid, Spain). Using control optical density OD values (OD_CTRL_) and test OD values (OD_TEST_), the agent concentration that caused 50% growth inhibition (IC_50_ value) was calculated from extrapolating in the trend line obtained by the formula (OD_CTRL_ - OD_TEST_)*100/OD_CTRL_.

### Fatty Acid Synthase Activity Assay

Cells were plated out at a density of 1x10^5^ cells/500 μL/well in 24-well microtiter plates. Following overnight cell adherence media was replaced by DMEM supplemented with 1% lipoprotein deficient Fetal Bovine Serum (Sigma) along with the corresponding IC_50_ concentrations of C75 (72 μM) and EGCG (265 μM) or DMSO. For the last 6 h of the treatment, ([1,2-^14^ C] Acetic Acid Sodium salt (53,9 mCi/mmol) (Perkin Elmer Biosciences, Waltham, MA, USA) was added to the media (1 μCi/mL). Cells were harvested and washed twice with phosphate-buffered saline (PBS) (500 μL) and once with Methanol:PBS (2:3) (500 μL). The pellet was resuspended in 0,2 M NaCl (100 μL) and broke with freeze-thaw cycles. Lipids from cell debris were extracted by centrifugation (2000 g, 5 min) with Chloroform:Phenol (2:1) (350 μL) and KOH 0,1 M (25 μL). The organic phase recovered is then washed with Chloroform:Methanol:Water (3:48:47) (100 μL) and evaporated in a Speed-vac plus SC110A (Savant). The dry-pellets were resuspended in ethanol and transferred to a vial for radioactive counting.

### Mitochondria Isolation of A549 Cells

Cells were grown to confluence in 10 mm dishes and collected in PBS (100 μL/dish). The pellet was resuspended in Buffer A (150 mM KCl, 5 mM Tris–HCl, pH 7.2) (125 μL/dish), and disrupted using a glass homogenizer (10 cycles with tight fitting pestle and 10 cycles with light one). Mitochondria were collected by centrifugation (16000 g, 5 min at 4°C), resuspended in Buffer A and quantified using Bradford-based Bio-Rad assay (BioRad Laboratories, Hercules, CA, USA). At this step mitochondria could be used for total CPT activity measurement.

### Carnitine Palmitoyltransferase (CPT) Activity Assay

CPT activity was assayed by the forward exchange method using L- [methyl-^3^ H] Carnitine hydrochloride (82 Ci/mmol) (Perkin Elmer Biosciences) as we previously described [25]. Briefly, reactions (were performed in the standard enzyme assay mixture (1 mM L-^3^ H]carnitine (~5000 dpm/nmol), 80 μM palmitoyl-CoA (Sigma), 20 mM HEPES (pH 7.0), 1% fatty acid-free albumin (Roche Sciences, Mannheim, Germany), 40–75 mM KCl and the corresponding IC_50_ concentrations of C75 (72 μM) and EGCG (265 μM) or DMSO when indicated. Reactions were initiated by addition of A549 isolated mitochondria (100 μg) and all incubations were done at 30°C for 3 min. Reactions were stopped by addition of 6% Perchloric Acid and then the product ^3^ H]-palmitoylcarnitine was extracted with butanol at low pH and was transferred to a vial for radioactive counting.

### Western Blot Analysis of Tumour and Cell Lysates

The primary mouse monoclonal antibody for FASN was from Assay designs (Ann Arbor, MI, USA). Monoclonal anti–β-actin mouse antibody (clone AC-15) was from Santa Cruz Biotechnology Inc. (Santa Cruz, CA, USA). Rabbit polyclonal antibodies against poly-(ADP-ribose)-polymerase (PARP), AKT, phospho-AKT^Ser473^, ERK 1/2, EGFR, phospho-EGFR^Tyr1068^, mTOR, phospho-mTOR^Ser2448^ and mouse monoclonal antibody against phospho-ERK1/2^Thr202/Tyr204^, were from Cell Signaling Technology, Inc (Danvers, MA, USA). A549 cells were harvested following treatment of A549 cells with EGCG or C75. Tumour tissues were collected from A549 human lung cancer xenografts at the end of the *in vivo* experiment. Cells and tumour tissues were lysed with ice-cold in lysis buffer (Cell Signaling Technology, Inc.) containing 1 mM EDTA, 150 mM NaCl, 100 μg/mL PMSF, 50 mM Tris–HCl (pH 7.5), protease and phosphatase inhibitor cocktails (Sigma). Protein content was determined by the Lowry-based Bio-Rad assay (BioRad Laboratories). Equal amounts of protein were heated in LDS Sample Buffer and Sample Reducing Agent from Invitrogen (California, USA) for 10 min at 70°C, separated on 3% to 8% or 4% to 12% SDS-polyacrylamide gel (SDS-PAGE) and transferred to nitrocellulose membranes. After blocking, membranes were incubated overnight at 4°C with the corresponding primary antibody. Blots were washed in PBS-Tween, incubated for 1 hour with corresponding peroxidase-conjugated secondary antibody and revealed using a commercial kit (Super Signal West Pico or Super Signal West Femto chemiluminescent substrate from Thermo scientific (Illinois, USA) or Immobilon Western HRP Substrate from Millipore (Massachusetts, USA)). Blots were re-proved with an antibody against β-actin as control of protein loading and transfer.

### *In vivo* Studies: Human Lung Tumour Xenograft and Long-term Weight Loss Experiments

Experiments were conducted in accordance with guidelines on animal care and use established by Biomedical Research Institute of Bellvitge (IDIBELL) Institutional Animal Care and Scientific Committee (AAALAC unit 1155). Tumour xenograft were established by subcutaneous injection of 10 x 10^6^ A549 cells mixed in Matrigel (BD Bioscience, California, USA) into 4–5 week old athymic nude BALB/c female’s flank (Harlan Laboratories, Gannat, France). Female mice A549 (12 wk, 23–25 g) were fed ad libitum with a standard rodent chow and housed in a light/dark 12 h/12 h cycle at 22°C in a pathogen-free facility. Animals were randomized into three groups of five animals in the control and four animals in the C75 and EGCG-treated groups. When tumours’ volume were palpable (reached around 35–40 mm^3^) each experimental group received an i.p. injection once a week of C75 or EGCG inhibitor (40 mg/kg) or vehicle alone (DMSO), dissolved in RPMI 1640 medium. Tumour volumes and body weight were registered the days of treatment and four days after every treatment until 33 days after first administration. Tumours were measured with electronic calipers, and tumour volumes were calculated by the formula: π/6 × (v1 × v2 × v2), where v1 represents the largest tumour diameter, and v2 the smallest one. At the end of the experiment, all mice were euthanized and tumour tissues were collected.

### Statistical Analysis

*In vitro* results were analysed by Student’s *t*-test or by one-way ANOVA using a Bonferroni test as a post-test. All data are mean ± standard error (SE). All observations were confirmed by at least three independent experiments. *In vivo* drug efficacy experiment results were analyzed using the non-parametric Wilcoxon test comparing repeated measurements (tumour volume). Data are the median of tumour volume of 4 or 5 animals. Statistical significant levels were p < 0.05 (denoted as *) and p < 0,001 (denoted as **).

## Results

### Effect of EGCG and C75 on FASN and CPT Activities in A549 Cells

**Figure 1:**
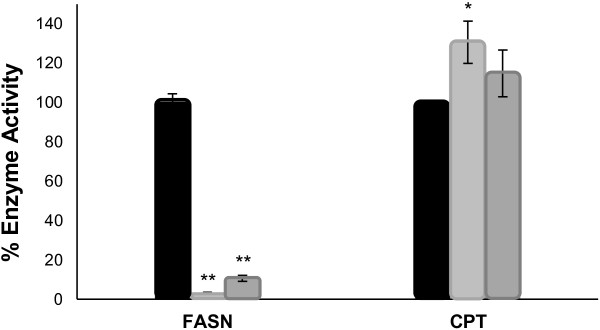
**EGCG inhibits FASN activity in A549 cancer cells with no change on CPT system activity.** A549 Cells were treated for 24 hours with C75 (72 μM) and EGCG (265 μM) and FASN activity was assayed by counting radiolabelled fatty acids synthesized *de novo*. Isolated mitochondria from A549 cells were assayed for CPT activity in the presence of DMSO (control), C75 (72 μM) or EGCG (265 μM), as described in Material and Methods. Bars represent the remaining enzyme activity in A549 treated cells or mitochondria. Data are means ± SE from at least 3 separate experiments. ** p < 0,001 versus control, by one-way ANOVA or Student’s *t*-test.

**Figure 2:**
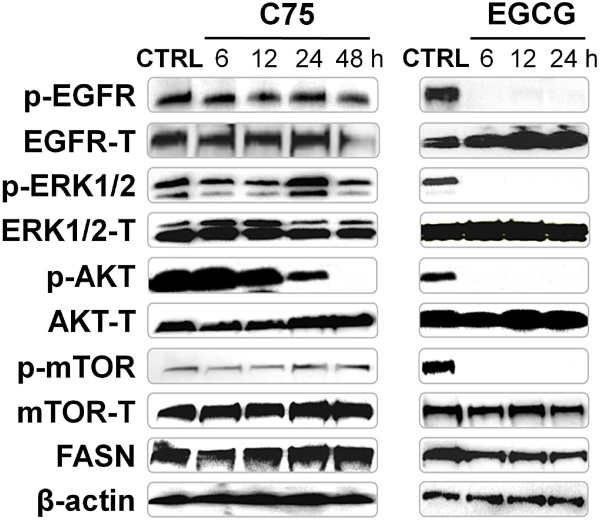
**EGCG blocks phosphorilation of EFGR, HER2, ERK1/2, AKT and mTOR in A549 cells.** A549 cells were treated for 6, 12, 24 and 48 hours with C75 (72 μM) and 6, 12 and 24 hours with EGCG (265 μM), and equal amounts of lysates were immunoblotted with anti-EGFR, anti-HER2, anti-ERK1/2, anti-AKT, anti-mTOR and anti-FASN antibodies. Activation of the protein under study was analyzed by assessing the phosphorylation status using the corresponding phospho-specific antibody. Total amounts of HER2 and AKT proteins remain unchanged. Blots were reproved with an antibody for β-actin to control for protein loading and transfer. Gels shown are representative of those obtained from three independent experiments.

In order to evaluate the specificity of EGCG and C75 for FASN, we analyzed their effect on FASN and CPT system activities. A549 cells were treated for 24 hours with IC_50_ concentration values of C75 (72 ± 2,8 μM) or EGCG (265 ± 7,1 μM) [ Additional file 1: Figure S1]. As shown in Figure 1, C75 and EGCG significantly reduced FASN activity in A549 cells compared to control cells (remaining FASN activity of 3,1 ± 0,6% and 10,7 ± 1,5%, p = 0,000; both). Significant changes in FASN protein levels were also observed in EGCG-treated cells but not in control or C75-treated cells, as assessed by Western blotting (Figure 2). The effect of both compounds on CPT enzymatic activity was assayed in A549 isolated mitochondria, as described in the Material and Methods section. EGCG had no effect on CPT activity (115 ± 12%, respect to control; p = 0,006), in contrast to C75, which produced a significant activation of CPT system (131 ± 11%, respect to control; p = 0,294).

### Analysis of the Effect of EGCG and C75 on Apoptosis and Cell Signaling in A549 Cells

**Figure 3:**
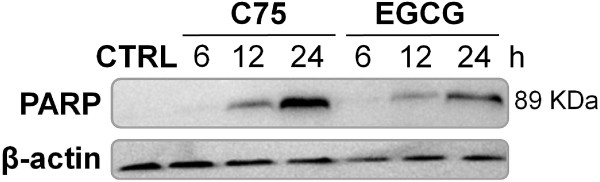
**EGCG and C75 induce apoptosis in A549 cells.** Induction of caspase activity was confirmed by PARP cleavage. A549 cells were treated with C75 (72 μM) or EGCG (265 μM) for 6, 12 and 24 hours, and equal amounts of lysates were immunoblotted with anti-PARP antibody, which identified the 89 kDa (cleavage product) band. Blots were reproved for β-actin as loading control. Gels shown are representative of those obtained from three independent experiments.

Apoptosis and induction of caspase activity were checked with cleavage of PARP in Western blotting analysis. Apoptosis was not detected in A549 non-treated cells. In A549 cells treated for 6, 12 and 24 hours with IC_50_ concentration values of C75 or EGCG ( Additional file 1: Figure S1), there was an increase in the levels of 89 kDa PARP product in a time-dependent manner (Figure 3). We examined the effects of EGCG and C75 on the phosphorylated and the total levels of EGFR (p-EGFR), HER2 (p-HER2), HER3 (p-HER3), HER4 (p-HER4) and its related downstream AKT, ERK1/2 and mTOR proteins. Results in Figure 3 confirmed that A549 cells treated with EGCG showed a marked decrease in the phosphorylated forms of EGFR, AKT, ERK1/2 and mTOR within 6 hours of EGCG treatment, with no changes in the total levels of the corresponding proteins. In contrast, C75 treatment needs up to 48 hours just to detect a partial decrease on total levels of EGFR protein and on p-AKT protein. Phosphorylated and total protein levels of HER2 (p-HER2), HER3 (p-HER3) and HER4 (p-HER4) did not change after C75- or EGCG-treatment (Data not shown).

### *In Vivo* Analysis of EGCG and C75 on Human Lung Cancer Xenografts

**Figure 4:**
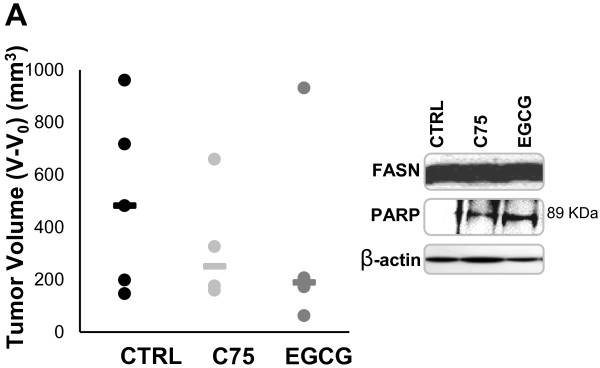
**EGCG inhibits A549 xenograft growth and do not induce *in vivo* weight loss.A**, once a week i.p. administration of 40 mg/kg of C75 (●) or EGCG (●) during 33 days blocked the growth of A549 lung cancer xenografts compared to control animals (●). Circles represent individual increase in tumour volume at final day (day 33) and horizontal lines represent the median value for each experimental group. C75 and EGCG-treated tumours showed apoptosis, whereas FASN protein levels did not change. Treated and control tumours were lysed and equal amounts of lysates were subjected to Western blot analyses with anti-PARP and anti-FASN. Blots were reprobed for β-actin as loading control. Gels shown are representative of those obtained from two independent experiments. **B**, EGCG treatment does not induce weight loss. The body weight of each mouse was measured before and weekly after treatment with C75 or EGCG (40 mg/Kg/day for 33 days) or vehicle control. Data are expressed as percentage of initial body weight and represent mean values ± SE for each experimental group.

To explore the potential effectiveness of EGCG and C75 for lung cancer treatment *in vivo,* we treated athymic nude mice with A549 human lung cancer xenograft. In control animals, on final day the median of the tumour volume (519 mm^3^ on day 33) was significantly different from the starting median tumour volume (33 mm^3^ on day 0, p = 0,04) and this trend (was similar from days 12 to 33 in control animals’ group (Data not shown). In the experimental animals, the median of the tumour volume of C75- and EGCG-treated animals on day 33 (290 and 224 mm^3^, respectively) wasn’t significantly different from the median of the tumour volume on the starting day (40 and 36 mm^3^, respectively; p = 0,07 both), those pointing out that the treatment with the anti-FASN compounds C75 and EGCG prevents the growth of A549 xenografts (Figure 4A). C75 and EGCG-treated tumours showed apoptosis by induction of PARP cleavage without any change in the total levels of FASN protein (Figure 4A). In EGCG-treated animals we do not find significant changes on fluid, food intake, body weight or other toxicity parameters (data not shown) versus control animals, after 33 days of weekly treatment with 40 mg/Kg of EGCG (Figure 4B). C75-treated animals showed a marked decrease of body weight (close to 6%) after each i.p. administration, which was especially remarkable in the first 20 days of treatment (Figure 4B).

## Discussion

Levels of FASN expression in different human carcinomas attracted considerable interest of this enzyme as a target for therapy [10,11]. In this study, we show that adenocarcinoma of lung cancer, is among the foremost of cancers that could potentially be treated by inhibiting FASN.

C75 has been studied in A549 lung cancer xenografts [28] where it induces a transient and reversible growth inhibition. EGCG anti-cancer effects in lung cancer have also been evidenced and, besides FASN-inhibition, several mechanisms of action have been proposed, such as G3BP1 (GTPase activating protein (SH3 domain) binding protein) inhibition [29], generation of Reactive Oxygen Species (ROS) [30] or induction of p53-dependent transcription [31].

To further investigate the implications of FASN inhibition in lung adenocarcinoma, we have analyzed the blockage of FASN by EGCG and C75 in A549 lung cancer cells. Firstly, we ensured similar levels of FASN inhibition by C75- and EGCG-treatment (96,9% and 89,3% of control, respectively). As C75 had no effect on the abundance of FASN protein levels and EGCG diminished the levels of this enzyme, it is probable that in the EGCG-treated cells, the reduction of FASN activity could be in part consequence of the reduced FASN protein levels.

The inhibition of FASN activity by EGCG and C75 was accompanied by an induction of apoptosis, and changes in cell growth and proliferation signaling pathways. The active phosphorylated form of EGFR (p-EGFR) was completely abolished after 6 hours of exposure to EGCG. Consequently, phosphorylated forms of ERK1/2 (p-ERK1/2), AKT (p-AKT) and mTOR (p-mTOR) were also markedly decreased. It is remarkable that comparable concentrations of C75, even with prolonged exposure (48 hours), only partially decreased total levels of EGFR and phosphorylated levels of AKT (p-AKT). Several data supported a relationship between HER2 and FASN in breast cancer, head and neck carcinomas, HER2-overexpressed fibroblasts and other carcinomas [11,32-35]. Furthermore, some authors have demonstrated the blocking effects of the FASN inhibitor EGCG on all members of epidermal growth factor receptor (ErbB) family [11,36-38].

This is the first evidence that EGFR is involved in the regulation of FASN expression in a lung cancer model with EGFR-overexpression. EGFR may be another EGCG-direct target that through inhibition of its downstream signalers (Akt, ERK1/2 and mTOR) is able to down-regulate FASN expression at two different levels: 1, at the transcriptional level through the sterol response element-binding proteins 1c (SREBP-1c), the FASN-transcription factor mediated by PI3K/Akt and MAPK/ERK1/2 pathways [39]; 2, at the translational level, through Akt-mTOR-signaling and its downstream effectors, eIF4G and S6K (reviewed in ref [40]) as seen in breast cancer [41] and in human hepatoma cells [42].

In addition, we corroborate a FASN-ErbB loop, described in breast cancer. The FASN disruption impedes synthesis of lipids, which are integrated in membrane lipid raft in which cell surface receptors, ErbB among others, accommodate and sense to tumourigenic pathways [43]. C75 is a direct and competitive inhibitor of FASN [21]. Consequently, we have seen a strong and fast inhibition of FASN activity with C75 treatment and a later effect on levels of EGFR and phosphorylation of it downstream effector Akt (p-Akt), what brings us to corroborate the idea of a FASN-lipid rafts-ErbB inhibition loop.

An important result of our study is the *in vivo* drug-efficacy study and long-term body weight evaluation. EGCG and C75 markedly blocked the growth of A549 lung cancer xenografts while the tumour volumes of control animals growth significantly until the final day study. C75-treated mice showed a marked decrease in body weight after each administration (close to 6% of initial body weight). This result accords to the data that C75 is able to stimulate CPT system and fatty acid β-oxidation, which has been related to the severe decrease of food intake and induction of weight loss in rodents [44]. In contrast, we have not observed a significant decrease in body weight in the animals treated for 33 days with EGCG.

A key feature of EGCG is that does not affect CPT activity (as it is shown in vitro in Figure 1) and, consequently, it does not induce weight loss in experimental animals. This result in a lung cancer model are in agreement with our previous findings in a mouse breast cancer model [11] and reinforces the hypothesis that CPT-activation is the cause of weight loss in xenografts models. Our data also reveal for the first time that the effects of EGCG in lung carcinoma involve different pathways than C75 but also that the undesirable side effects observed in C75 treated-mice are not produced in EGCG-treated mice.

## Conclusions

In conclusion, the work reported here supports the development of EGCG as a FASN inhibitor for adenocarcinoma lung cancer treatment. EGCG acts as potent and lipogenic-selective inhibitor of FASN, and do no exhibit adverse effects on body weight, therefore holding promise for further target-directed anti-cancer drug studies either alone or co-administered with other antitumoural drugs.

## Abbreviation

FASN: inhibition in lung cancer.

## Competing interests

None of the authors has any potential conflict of interest regarding this work.

## Authors’ contributions

JR carried out the activity assays, participated in the design of the study, performed the statistical analysis and drafted the manuscript. AB carried out the immunoassays, performed the statistical analysis and drafted the manuscript. GO carried out the immunoassays. SC carried out the *in vivo* assays. TP conceived of the study and drafted the manuscript. TP, DH and PM participated in the design and coordination of the study. All authors have approved the final version of the manuscript.

## Pre-publication history

The pre-publication history for this paper can be accessed here:

http://www.biomedcentral.com/1471-2407/12/280/prepub

## Supplementary Material

Additional file 1 **Figure S1. EGCG and C75 show cytotoxic activity in A549 human lung carcinoma cells.** A549 cells were treated with different concentrations of C75 (20 – 200 μM) or EGCG (40 – 300 μM) for 48 hours. Pale gray (●) and dark grey (●) circles represent the percentage of A549 cell proliferation inhibition after C75 and EGCG treatment respectively, which was determined using an MTT assay. Results are expressed as mean percentage of inhibition in cell proliferation from three independent experiments performed in triplicate ± SE. PDF File Format. Click here for file
