# Supplementary figures and images for: Different fatty acid metabolism effects of (−)-Epigallocatechin-3-Gallate and C75 in Adenocarcinoma lung cancer
Source: BMC Cancer. 2012 Jul 6;12:280. doi: 10.1186/1471-2407-12-280 (PMC3500220; doi:10.1186/1471-2407-12-280)

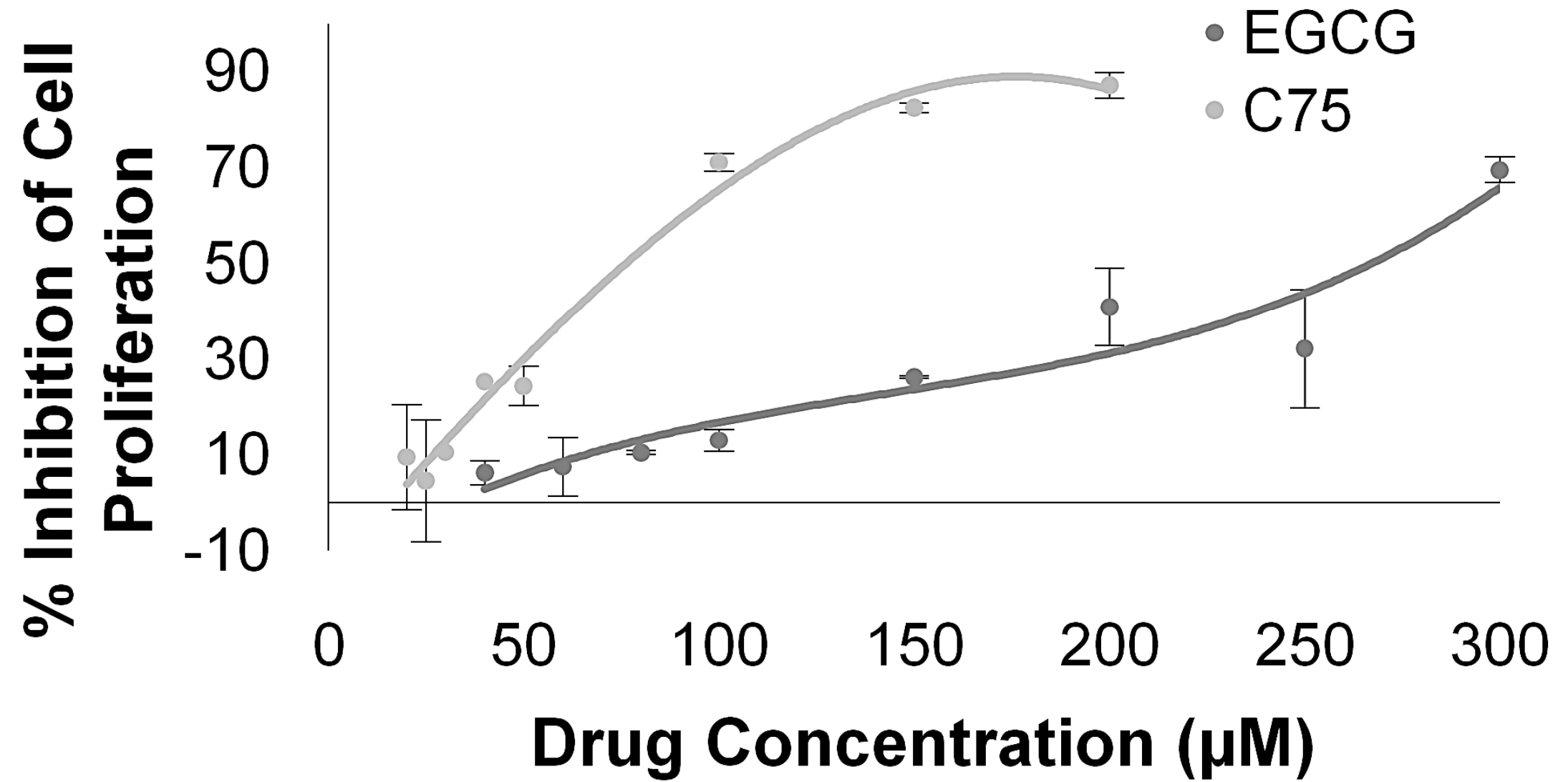

Supplement: Additional file 1 — Figure S1. EGCG and C75 show cytotoxic activity in A549 human lung carcinoma cells. A549 cells were treated with different concentrations of C75 (20 – 200 μM) or EGCG (40 – 300 μM) for 48 hours. Pale gray (●) and dark grey (●) circles represent the percentage of A549 cell proliferation inhibition after C75 and EGCG treatment respectively, which was determined using an MTT assay. Results are expressed as mean percentage of inhibition in cell proliferation from three independent experiments performed in triplicate ± SE. PDF File Format. [file 1471-2407-12-280-S1.pdf]
